# Quality of Eggs, Concentration of Lysozyme in Albumen, and Fatty Acids in Yolk in Relation to Blue Lupin-Rich Diet and Production Cycle

**DOI:** 10.3390/ani10040735

**Published:** 2020-04-23

**Authors:** Emilia Kowalska, Joanna Kucharska-Gaca, Joanna Kuźniacka, Lidia Lewko, Ewa Gornowicz, Jakub Biesek, Marek Adamski

**Affiliations:** 1 Department of Animal Breeding, Faculty of Animal Breeding and Biology, UTP-University of Science and Technology in Bydgoszcz, Mazowiecka 28, 85-084 Bydgoszcz, Poland; emilia.kowalska22483@wp.pl (E.K.); joasia8929@wp.pl (J.K.-G.); kuzniacka@utp.edu.pl (J.K.); adamski@utp.edu.pl (M.A.); 2 Water Poultry Genetic Resource Station in Dworzyska, Kołuda Wielka Experimental Station, Institute of Animal Production - National Research Institute, 32-065 Kórnik, Poland; lidia.lewko@izoo.krakow.pl (L.L.); ewa.gornowicz@izoo.krakow.pl (E.G.)

**Keywords:** laying hens, narrow-leafed lupin, peas, egg quality, lysozyme, fatty acids

## Abstract

**Simple Summary:**

Eggs are an integral part of many people’s diets. Laying hens are commonly fed on soybean meal, which is often genetically modified. It is possible to replace soybean in feed with other high-protein plants. Legumes, including lupins and peas, have the potential to be used in poultry nutrition. The quality of eggs for consumption depends on nutrition and the age of the laying hens. In our research, the goal was to assess the quality of eggs, including the content and activity of lysozyme and the content of fatty acids in egg yolk, depending on the provision of feed with 10%, 15%, 20% or 25% narrow-leafed (blue) lupin (cultivar Boruta) and 10% pea (cultivar Muza) during the laying period. The results show that feeding with lupin had a positive effect on egg yolk saturation, which is important to consumers. A beneficial effect of the proposed feed on the profile of omega-6 and -3 fatty acids and hypocholesterolemic acids was also found. In almost all proposed diets, there was no negative impact of the use of lupins on the weight and physical characteristics of eggs or the characteristics of lysozyme. Changes in egg quality during the laying period are associated with natural changes in the laying physiology of hens. The use of narrow-leafed lupins and pea seeds could be proposed as an alternative to soybean meal for laying hens in countries where the environmental conditions are not good for soybean production. This would offer a wider range of choices in the consumer market since, nowadays, products from animals raised on GMO feeds are not preferred.

**Abstract:**

In recent years, the interest in lupin seeds as a source of protein in poultry nutrition has increased. The aim of this study was to assess the quality of table eggs produced by hens that were fed diets containing pea seeds and various levels of narrow-leafed lupin as a substitute for soybean meal. The share of lupin seeds in the treatment groups was 10%, 15%, 20% and 25%. Egg morphology, the fatty acid profile in egg yolk and the amount and activity of lysozyme in egg white were analysed. Results show that using 10–20% lupin seeds in feed in the diet of laying hens in intensive farming does not result in a change in weight or egg structure, their physical properties or their morphological composition. Increasing the share of lupin seeds in feed for laying hens increases the saturation of the colour of egg yolks, which is a desirable feature among consumers. The use of lupin seeds in feed for laying hens does not adversely affect the chemical properties of egg proteins, as expressed by the amount and activity of lysozyme. In feed for laying hens, replacing soybean meal with lupin seeds has a positive effect on the fatty acid profile in egg yolk (omega-3 and -6 polyunsaturated acids and hypocholesterolemic acids).

## 1. Introduction

Laying hen nutrition is essential to achieve the best egg production and to maintain the good health of the flock, and special attention is paid to the source, content and quality of protein in feeds [1]. Soybean meal (SBM), currently the most popular component of feeds, is characterised by a high content of protein, low content of antinutrients and beneficial composition of amino acids [2]. White, narrow-leafed and yellow lupins, as well as faba bean, pea and rapeseed, could also be used as protein-rich feed components of plant origin [3,4]. Kaczmarek et al. [3] reported that protein from lupins and SBM are utilised to the same degree. In the past, the use of lupins and other legume seeds was considerably limited because of the high content of antinutrients (alkaloids, non-starch polysaccharides (NSP)), whose level depends on the plant variety and growing conditions. However, new varieties of lupins have been created that are characterised by high protein and reduced levels of alkaloids [5,6,7]. Several studies have investigated the use of lupins in the diets of laying hens (Hy-Line Brown). Rutkowski et al. [8] tested diets with graded inclusion (10%, 15%, 20%, 25%) of yellow lupin (variety Mister) and constant 10% inclusion of peas (var. Trachalska). The authors concluded that laying hens’ diets that contained up to 20% yellow lupin had no negative effect on egg production or egg weight, and yolk colour was more saturated when the diet contained greater amounts of lupin. Similar findings were reported by Hammershøj and Steenfeldt [1], who investigated the effects of 15% inclusion of blue lupin (narrow-leafed lupin) on the quality of hen eggs. Rutkowski et al. [7] found that the use of about 19.48% lupin seeds, peas and rapeseed meal (including 22% narrow-leafed lupin) could be accepted as an SMB substitute in the diet of laying hens.

Egg quality is an important aspect of poultry production and influences the profitability of production and consumer satisfaction. Many factors determine egg quality, including genotype, age of birds, management system and diet [9]. Egg quality parameters include egg morphology and components, egg weight and shape, and the proportion of yolk, albumen and shell. Another important aspect is egg freshness, as measured by the height of the thick albumen and Haugh unit (HU) score [10]. Consumers pay much attention to yolk colour, which mainly depends on diet and the assimilation of xanthophylls (carotenoids) from the feed [8,11]. Eggs can also be used in human and veterinary medicine because they are rich in bioactive substances [12]. Lysozyme is an alkaline globular protein with antibacterial properties. It can destroy bacterial cell walls [13]. In addition, the fats contained in egg yolk have a particularly high biological value because of their beneficial unsaturated acid/polyunsaturated fatty acid (UFA/PUFA) ratios and the high content of valuable phospholipids. Palmitic acid (SFA) found in eggs exerts a hypercholesterolemic effect, which is not beneficial to consumers. However, eggs also contain fatty acids with positive effects on human health such as oleic acid (C:18:1 n-9), linoleic acid (C:18:2), linolenic acid (C18:3 n-3) and docosahexaenoic acid (C:22:6 n-3) [14].

Tested hypothesis: diets with graded inclusion of narrow-leafed lupin and constant 10% addition of peas as a substitute for soybean meal influence the quality of eggs from Hy-Line Brown laying hens.

The aim of the study was to assess the quality of eggs for consumption depending on the use of graded inclusion of narrow-leafed seeds to replace soybean meal and the age of Hy-Line Brown laying hens.

## 2. Material and Methods

According to directive no. 2010/63 / EU and the resolution of the National Ethics Committee no. 13/2016 the consent of the Local Ethics Committee for this type of research is not required.

### 2.1. Bird Management 

Quality analysis was performed for 1950 eggs from Hy-Line Brown laying hens. Hens were allocated to 5 groups (A–E), with 3 hens in each cage. The area of the cage per one hen was 0.08 m^2^, according to the legal principles prevailing in Poland. The control group (A) received a diet based on soybean meal (SBM), and the other treatment groups (B–E) received diets with graded inclusion of narrow-leafed lupin variety Boruta (*Lupinus angustifolius* L., cv. Boruta) (B: 10%; C: 15%; D: 20%; E: 25%) with the addition of pea variety Muza (*Pisum sativum* L., cv. Muza) at the 10% level. Birds received feed and water ad libitum. Feeds contained 11.30 MJ/kg of metabolisable energy (ME) and 16.20% total protein. The composition and nutritional value of feeds for laying hens are presented in Table 1. Experimental diets were formulated to meet the requirement for Hy-Line Brown laying hens and were mixed in a commercial feed mill. The quality of the experimental diets and nutritive values were determined and provided by the feed producer. Egg quality was analysed between weeks 2 and 33 of egg production at 2.5-week intervals (periods I–XIII). When the 2nd week of egg production began, hens were 20 weeks old. A single analysis was performed for 150 eggs (30 eggs per treatment) 24 h after egg collection. Eggs were collected randomly from the cages of the mentioned treatments and assigned a number from 1 to 30 in each group. This assignment allowed each egg to be treated as an individual unit for analyses.

### 2.2. Egg Quality

Each egg was a single unit in the quality assessment. Egg quality analysis included the determination of egg weight, yolk weight and weight of thick and thin albumen measured using RADWAG PS 750/X (Radwag, Radom, Poland) scales (± 0.01 g). The egg shape index (egg width/length) was calculated using measurements taken with a Mitutoyo Quantu Mike calliper (Mitutoyo, Wrocław, Poland). Eggshell surface area was calculated using the egg weight (W) with a formula proposed by Paganelli et al. [15]: P_s_ = 4.835 × W^0.662^. Eggshell strength (kg/cm^3^) was analysed with an Egg Force Reader (Orka Food Technology Ltd., Toruń, Poland). Thick albumen height was measured with a QCD device (TSS, Poznań, Poland). Yolk colour was assessed using the 15-tone La Roche scale. Yolk colour was also assessed using a Konica Minolta colourimeter, model CR400, Japan. The device was calibrated using the white calibration plate no. 21033065 and the D_65_ Y_86.1_ x_0.3188_ y_0.3362_ scale. Colour was graded according to the CIE (1986) L*a*b* system (L* = lightness, a* = redness and b* = yellowness). Haugh unit (HU) scores were calculated from the formula HU = 100 lg (H + 7.7 – 1/7W ^0.37^), where H is the height of the thick albumen (mm) and W is the weight of the egg (g) [16]. The specific density of thick albumen and yolk was determined using KIT-128 (for the analysis of the density of liquids and solids, Radwag, Radom, Poland) and RADWAG 750/X scales. Shells from broken eggs were collected on trays and dried for 3 hours at 105 °C in a SUB 100M drying chamber (Binder, Tuttlingen, Germany). After drying, the shells were weighed (RADWAG PS 750/X) and measured for thickness with a screw thread micrometre (TSS, Poznań, Poland). The proportion of morphological components of the egg was calculated.

### 2.3. Lysozyme and its Activity in Albumen

Thick and thin albumen (10 samples from each group and on each date) were collected in sterile containers before analysis. The concentration (%) and hydrolytic activity of lysozyme were analysed using an SP-830 plus spectrophotometer Metertech (Merazet, Poznań, Poland) and a technique described by Adamski et al. [13] based on the Leśnierwski and Kijowski [17] method, which relies on the lysis of bacterial cell walls in *Micrococcus lysodeikticus*. The hydrolytic activity of lysozyme was expressed in units (U), assuming that one unit of lysozyme will produce a ΔA_450_ of 0.001 per minute at pH 6.24 at 25 °C using a suspension of *Micrococcus lysodeikticus* as the substrate in a 2.6 mL reaction mixture. The reaction mixture contained 0.1 mL of lysozyme solution + 2.5 mL of bacterial suspension and was placed in a cuvette (light path = 1 cm). After calculating the decrease in absorbance (ΔA) for the working lysozyme solution, the curve of absorbance versus enzyme concentration was plotted. Lysozyme activity in the tested sample was determined from the reference curve. A decrease in absorbance for the solution (ΔA) was calculated using the following formula: ΔA = A_t0_ – A_t_ (U/min), where A_t0_ is the absorbance of the bacterial suspension at time t_0_, and At is the absorbance of the bacterial suspension after time t.

### 2.4. Fatty Acid Profile in the Yolk

The fatty acid profile in egg yolks was analysed at the beginning, at the peak and at the end of the egg production period. Five yolks were sampled from each treatment group and on each date. The yolks were collected in sterile containers, frozen at −18 °C and freeze-dried in an Alpha plus freeze dryer (Donserv). Fat was extracted from yolks using a technique proposed by Folch et al. [18], with a mixture of chloroform and methanol (2:1 v/v) and a shaker. The samples were filtered and left for 24 h for evaporation. Fatty acid methyl esters were prepared according to the PN-EN ISO 12966-2 standard (2011) in the following order: fat dissolution in isooctane, transmethylation with potassium hydroxide solution in methanol, neutralisation of potassium hydroxide with acidic sodium sulphate, salting out of esters with sodium chloride solution.

Saponified fatty acid esters were separated on a 7890 B gas chromatograph (Agilent Technologies, Perlan Technologies, Warszawa, Poland) with an MSD 5977A detector and an autosampler. A capillary column (DB-225 MS, 60 m × 0.25 mm × 0.25 µm) was used for analysis. Analytical parameters were as follows: injection port temperature (split mode 1:100): 230 °C; transfer line temperature: 230 °C; ion source temperature: 230 °C; quadrupole temperature: 150 °C; mode: SIM (selected-ion-monitoring); ionisation type: EI (electron impact). Oven temperature settings were 70 °C with an increase of 0.0 °C/min; hold time of 0.0 min; 210 °C with an increase of 7.0 °C/min; hold time of 65.0 min. The carrier gas was helium. The flow rate was 1.0 mL/min; the volume of the injected sample was 1.0 µL. Fatty acid methyl esters were identified using the Supelco 37 standard FAME Mix component.

### 2.5. Analytical Methods

For chemical analyses, representative samples of seeds were ground to pass through a 0.5 mm sieve. Narrow-leafed lupin seeds and pea seeds were analysed in duplicate for crude protein (CP) and ether extract (EE) (methods 976.05, 920.39, respectively, according to Association of Official Agricultural Chemists (AOAC) [19] procedures). In addition, acid detergent fibre (ADF, expressed together with residual ash) and neutral detergent fibre (NDF) with heat-stable amylase and expressed together with residual ash were analysed in seeds (methods 942.05, 973.18, respectively, according to AOAC [19]). Starch content in peas was determined using an analytical kit specific for the agricultural industry (Megazyme International; AOAC, 2005: Method 996.11) based on heat-stable α-amylase and amyloglucosidase. Amino acid (AA) content was determined in an AAA-400 automated amino acid analyser using ninhydrin for post-column derivatisation (procedure 994.12; AOAC [19]). The content of tannins in a pea sample was analysed according to the technique proposed by Kuhla and Ebmeier [20]. Raffinose family oligosaccharides (RFO) were extracted and analysed by high-resolution gas chromatography. Phytate was determined according to the technique proposed by Haug and Lantzsch [21]. Lupin alkaloids were extracted from flour with trichloroacetic acid and methylene chloride (SigmaAldrich, Munich, Germany). Alkaloids were determined by gas chromatography (GC) (Shimadzu GC17A, Kyoto, Japan) on a capillary column (Phenomenex, Torrance, USA).

### 2.6. Statistical Analysis

Data were processed using Statistica 12.5 PL software (2007). Mean values for all analysed parameters and their standard deviations (± SD) and coefficients of variation (v) were calculated. A two-way model of ANOVA was used to analyse variability (variable 1: diet; variable 2: egg production period). The significance of differences was verified using the Tukey test. Interactions between experimental variables were assessed. The significance of differences was adopted at *P* ≤ 0.05.

## 3. Results

### 3.1. Chemical Composition of Narrow-Leafed (blue) Lupin and Pea Seeds

Analysis of chemical composition demonstrated that narrow-leafed lupin seeds contained 88.62% dry matter (DM), 36.88% crude protein and 15.09% fibre. Narrow-leafed lupin seeds contained 21.43% ADF and 25.92% NDF. The energy value of narrow-leafed lupin seeds was 20.73 MJ/kg. The content of antinutrients, i.e., oligosaccharides, raffinose and P-phytate, was 8.77 g/kg DM, 1.20 g/kg DM and 0.42 g/kg DM, respectively. Narrow-leafed lupin seeds were also analysed for the content of amino acids (39.39 g/kg DM), minerals and alkaloids (440 mg/kg). However, pea seeds had 86.65% dry matter and 27.57% crude protein. Lupin seeds are known for not containing starch; thus, the starch assay was not performed on the test ingredient. Pea seeds contained 44.23% starch. There was also lower content of ADF and NDF than in lupin seeds. Pea seeds are a good addition to diets because of the lack of alkaloids, but the amount of total oligosaccharides is similar in both species of legumes. Detailed data for all parameters are presented in Table 2.

### 3.2. Weight, Shape and Surface Area of Eggs

The weight and surface area of eggs were highest (63.79 g and 73.65 cm^2^) in group B, fed a diet with 10% inclusion of narrow-leafed lupin, and the lowest values were in group E, fed a diet with 25% inclusion of lupin (61.27 g and 63.65 cm^2^) (*P* < 0.05). The highest weight and surface area of eggs were found on dates IX (66.28 g, 77.65 cm^2^), XII (65.37 g, 76.89 cm^2^) and XIII (65.78 g, 77.22 cm^2^) compared with date I (53.52 g, 67.36 cm^2^), and the differences were significant. The egg width-to-length ratio (the egg shape index) did not differ significantly between treatment groups (*P* > 0.05), but the highest (significant at *P* < 0.05) values were found on dates I–III (78.82–78.88). After that, a significant decrease in the egg shape index occurred gradually until date XIII (76.04). There was a significant (*P* < 0.05) interaction between both experimental variables and the egg shape index (Table 3).

### 3.3. Eggshell

The share of narrow-leafed lupins in hens’ diets had no significant effect on the strength and thickness of the eggshell (*P* > 0.05). The weight of the eggshell and its proportion in the egg was highest in group A, fed an SBM-based diet (6.31 g; 9.99%), and lowest in groups C and E (6.04 g), fed diets with 15% and 25% inclusion of lupin, respectively. The proportion of shell in the egg was lowest in groups B (9.64%) and C (9.65%) (*P* < 0.05). Eggshells collected on date IV were strongest (4.61 kg/cm^2^) compared with those collected on other dates (*P* < 0.05). Significant increases in the proportion of eggshell and its thickness were found on the last three dates (XI–XIII) (*P* < 0.05), but the thickness of eggshell on all dates of quality assessment was comparable. There was a significant (*P* < 0.05) interaction between diet and the egg production date for almost all egg parameters other than eggshell thickness (Table 4).

### 3.4. Egg Components

The weight and proportion of thick albumen were highest in group D, fed a diet with a 20% inclusion of narrow-leafed lupin (22.93 g, 36.35%). The weight of thin albumen, total albumen and its proportion in the egg were highest in group B (21.35 g, 33.45% and 43.33 g, 67.92%, respectively), fed a diet with a 10% inclusion of lupin (significant at *P* < 0.05). Yolk weight was highest in the control group (A: 14.44 g) compared with group E (14.07 g), and a significant difference *(P* < 0.05) was found for the proportion of yolk in the egg between groups E (22.92%) and B (22.46%). Graded inclusion of narrow-leafed lupin in hens’ diets had no significant effect on the height of thick albumen or Haugh unit score *(P* > 0.05). There were significant differences in the weight of egg components between egg production dates, and the total albumen weight and weight of yolk were significantly higher in the middle and at the end of the egg production period *(P* < 0.05). The proportion of thick albumen and yolk were highest on the last study dates, while the proportion of total albumen was highest on dates I–II *(P* < 0.05). The height of thick albumen decreased towards the end of the egg production period. A similar trend was found for the Haugh unit score *(P* < 0.05) (Table 5). A significant interaction between variables was found for almost all analysed egg components, except the weight and proportion of yolk *(P* > 0.05).

### 3.5. Yolk Colour

The subjective assessment of yolk colour using the 1–15 scale (La Roche) showed significantly higher colour saturation in groups D and E (8.40 and 8.54, respectively) than those in group B (7.51) *(P* < 0.05). There were significant differences in colour between subsequent egg production dates (I–V: 11.37–12.59 versus VI–XIII: 4.27-5.27) *(P* < 0.05). The objective analysis of yolk colour using the CIE L*, a*, b* system revealed the highest (significant at *P* < 0.05) yolk lightness (L*) in groups A and B (48.64 and 48.39, respectively) and on dates XI–XIII (54.39–53.97), while redness and yellowness were the lowest in these groups (a* − 1.72, −1.20; b* 17.65, 21,82). Yolk redness decreased on subsequent dates (from 4.62 to − 6.11), and yellowness was the highest (33.72) on date XIII *(P* < 0.05). Yolk colour assessed using the La Roche scale and L*, a*, b* system depended on a significant interaction *(P* < 0.05) between diet and egg production date (Table 6).

### 3.6. Lysozyme Concentration and Activity in Albumen

The concentration and activity of lysozyme in thick albumen were significantly higher *(P* < 0.05) in the control group (A), fed an SBM-based diet (0.210%, 44,569 U/mg), than in group B, fed a diet with 10% inclusion of narrow-leafed lupin (0.190%, 40,577 U/mg). The coefficient of variation (V) for the content of lysozyme and its activity for thick albumen was 20.25% and 20.16%, respectively, and 11.24% and 13.11% for thin albumen. However, the thin albumen obtained from eggs from group E, fed a diet with 25% inclusion of narrow-leafed lupin, had the highest lysozyme concentration (0.452%) and activity (96,165 U/mg) *(P* < 0.05). The concentration and activity of lysozyme were 0.214% and 45,574 U/mg in thick albumen on date IV and 0.486% and 103,313 U/mg in thin albumen on date III. Lysozyme concentration and activity were significantly lower on other egg production dates *(P* < 0.05). The coefficient of variation calculated for the egg production date was similar in all treatment groups (thick albumen: 19.27%, 19.20%; thin albumen: 10.27%, 11.58%, respectively). An interaction between variables was found in all aspects of the lysozyme assay and its activity *(P* < 0.05) (Table 7).

### 3.7. Fatty Acids in Egg Yolk

Analysis of the data presented in Table 8 revealed that the lowest content of C15:0, C17:0 and C18:2n6 was in the egg yolk from control hens that were fed a diet without narrow-leafed lupin (significant at *P* < 0.05). Egg yolks from groups D and E were characterised by a lower content of C16:1 but a higher content of C17:0 and C18:2n6 compared with group A *(P* < 0.05). Fatty acid content differed significantly between egg production dates, but no clear conclusions could be reached. For some fatty acids, the highest levels were found mainly on the first egg production date (I). For the content of C14:0, C16:0, C18:0, C18:1n9, C18:3n3, C20:2n6 and C22:0 in egg yolk, there was an interaction between the type of diet and egg production date *(P* < 0.05).

## 4. Discussion

The present experimental study analysed the effect of diet on the quality of eggs and the effect of hen age on the morphological traits of eggs. Many authors [22,23,24] have reported a significant influence of hen age on egg weight, but Zemkova et al. [25] found no correlation between these parameters. 

Other studies [23,24,26] have revealed that the age of hens correlated with a higher proportion of yolk in eggs and a lower proportion of albumen in eggs. According to Silversides and Scott [27], the age of hens influences the quality of eggshell. On the other hand, Van den Brand et al. [24] found that the age of hens did not affect eggshell thickness but was associated with a lower egg shape index. A study on laying hens (Lohmann Brown) managed in a cage system and fed diets with 10% and 20% inclusion of narrow-leafed lupin per feed ration also found no significant reduction in the weight of eggs during the laying period (weeks 4–20) [28]. Consistently, Park et al. [29] found no significant effect of the egg production period and diet (LAn11, 16.5, 22) on the weight of eggs from Hy-Line Brown hens. Our research, carried out on eggs from Hy-Line Brown hens, revealed that the weight of eggs differed between groups that were fed a lupin-based diet and controls that were fed an SBM-based diet. Laudadio and Tufarelli [30] reported no significant effect of various sources of vegetable protein in feed on egg weight. In a study by Lee et al. [31], 15% inclusion of whole or dehulled narrow-leafed lupin seeds (LAn) had no negative effect on the weight of eggs from hens (*Gallus gallus domesticus* L.; Bevan Brown). Hammershøj and Steenfeldt [1] reported a negative effect of blue lupin seeds (15%; 25%) as a replacement for SMB on the weight of eggs from hens that were managed in the organic system. In addition, the weight of eggs increased with the age of hens (ISA Brown) between weeks 20 and 31 in all treatment groups (SBM, *Lupinus angustifolius*: LAn15 and 25).

The effects of a diet containing yellow lupin seeds (*Lupinus lutues:* LL10, 15, 20 and 25) on the quality of eggs from Hy-Line Brown hens were investigated by Rutkowski et al. [8]. They reported no significant correlation between different protein sources and the weight of eggs in weeks 1–5, 20 and 21 of egg production. However, in weeks 6–10 and 12 of egg production, the weight of eggs was significantly lower in birds fed a diet with 25% inclusion of yellow lupin seeds. Between weeks 14 and 16 of egg production, the alternative source of protein had a positive effect on the weight of eggs in group LL10. The heaviest eggs were found in the control group and groups LL20 and 25 in week 22 (63.10, 64.20 and 63.30 g, respectively), but the heaviest eggs in group LL10 were found in week 16 (65.70 g), and those in group LL15 were found in week 19 of egg production (65.10 g). In our study, the heaviest eggs were laid by Hy-Line Brown hens in weeks 23 (IX), 30 (XII) and 33 (XIII) of egg production (66.28, 65.37 and 65.78 g, respectively), and the lightest eggs were laid at the beginning of the study, in periods I–III (weeks 2–8). Another study [7] indicated that over a 17-week egg production period, the weight of eggs was highest (57.92 g) in control hens (SBM) compared with other treatment groups (55.94 g for LAn10 + LL2 + PS8 and 54.99 g for LAn10 + LL12 + PS5). Our study also revealed that the complete replacement of SBM with lupin seeds (LAn25) was associated with a reduction in egg weight *(P* ≤ 0.05). Koivunen et al. [32] reported a significant reduction in egg weight in hens that were fed diets with 5% and 10% inclusions of faba beans. They attributed this effect to the presence of vicine in faba beans, an antinutrient responsible for erythrocyte haemolysis. In addition, vicine may reduce the amount of precursor material in the granulosa cells and their activity, or they may destroy oocytes. On the other hand, Fru-Nij et al. [33] demonstrated that reduced egg weight could be associated with methionine and cysteine deficiency in faba beans.

When laying hens were fed a diet with faba bean meal, the proportion of shell in the egg decreased with the age of birds between weeks 43 and 63 of life. This could be the result of the abnormal metabolism of calcium and phosphorus and increased activity of vitamin D3 in plasma, resulting in calcium imbalance [34]. Blue lupin (LAn) in feed (10% and 20%) had a positive impact on the quality of eggs, shell thickness *(P* = 0.001), the proportion of shell in eggs *(P* = 0.002) and shell strength *(P* = 0.036) compared with eggs from the control group (SBM) [28]. Lee et al. [31] found no significant effect of whole and dehulled blue lupin seeds (LAn15) on the weight of eggshell. In our study, we found no significant differences in the thickness and strength of eggshell between treatment groups, which implies that 25% inclusion of narrow-leafed lupin in feed does not deteriorate the quality of eggshells. Consistent findings were reported by Krawczyk et al. [35] and Park et al. [29], who reported that the inclusion of *Vicia Faba* (VF)11, 16.5 and 22 in the diet of Hy-Line Brown hens did not reduce the strength of eggshells. Our study demonstrated that different diets had no effect on the strength or thickness of the eggshell. Laudadio et al. [36] reported that a diet with 15% inclusion of alfalfa did not worsen the quality parameters of eggshells from ISA Brown hens in terms of the thickness, strength and proportion of shell in the egg. Additionally, 10–100% inclusion of white lupin in feed had no negative effect on the weight or thickness of eggshells [37]. The strength of the eggshell decreases with the age of hens, which is associated with lower availability of calcium and phosphorus and with structural changes in the shell [38]. This was also confirmed by Drażbo et al. [28], who reported a lower thickness, strength and proportion of shell in the egg throughout the egg-laying period (from weeks 26 to 38). Similar but insignificant differences were found in a study that analysed eggs from Leghorn hens, and the strength and thickness of eggshells decreased with the age of hens (53–74 weeks of life), both in the control birds (SBM) and in treatment groups (VF5 and 10) [32]. The inclusion of lupin seeds in birds’ diets in our experiments was associated with improvements of these quality parameters. Mitsuoka [39] and Martínez-Villaluenga et al. [40] indicated that the oligosaccharides found in lupin seeds are a natural prebiotic that stimulates the proliferation of bifidobacteria in the colon, and the interaction of these bacteria with short-chain fatty acids increases the absorption rate of calcium, which is the main component of the eggshell. Laudadio and Tufarelli [30] found that a diet containing lupin seeds had no effect on the quality parameters of eggshell. Rutkowski et al. [7] reported a significant association between the egg production date and egg weight, the proportion of shell in the egg and shell thickness, measured in weeks 5 (5.30 g; 9.70%; 0.357 mm) and 13 (5.90 g; 9.50%; 0.366 mm). Differences in the analysed egg traits, both in the present study and studies reported by other authors, may result from the structure of the shell, particularly from the crystalline layer of the shell and the content of minerals [41].

Our study on Hy-Line Brown hens showed no significant effect of diet on the height of thick albumen or Haugh unit scores. This is consistent with findings by Koivunen et al. [32], who reported no significant effect of raw and processed faba beans (VF50 and 10) on the quality of albumen (HU) in laying hens (Leghorn). In addition, higher Haugh unit scores were found in each treatment group (SBM, VF) on subsequent dates of egg production (weeks 53–74 of life). On the other hand, Drażbo et al. [28] found no negative effect of diets containing narrow-leafed lupin seeds (LAn10 and LAn25) on these traits. Rutkowski et al. [7] reported a positive effect *(P* ≤ 0.05) of diets containing lupin seeds and peas (LL12 + LAn10 + PS5) on the quality of albumen (expressed in Haugh units) or the albumen index in Hy-Line Brown hens. Our study revealed a significant effect of birds’ diets on the morphological and physical parameters of eggs, consistent with reports by Rutkowski et al. [8]. The proportion of egg yolk (14.30 g) and thin albumen (19.10 g) was highest in eggs from Hy-Line Brown hens fed a diet with 15% inclusion of yellow lupin *(P* ≤ 0.05). The proportions of egg yolk, thin albumen and the weight of thick albumen were lowest in group LL25 (13.90, 17.80 and 21.40 g, respectively). Egg yolks were largest in the control group *(P* ≤ 0.05), but this quality parameter did not deteriorate in groups LAn10, 15 and 20. Rutkowski et al. [8] reported that the weights of yolk (14.07 g) and thick albumen (21.42 g) were lowest in hens that were fed a diet with 25% inclusion of an alternative protein source (LAn25). Drażbo et al. [28] demonstrated that a diet with the inclusion of lupin seeds had no effect on the proportion of yolk and albumen in eggs. Rutkowski et al. [7] also found that the inclusion of lupin seeds had no significant effect on the weight of yolk or the proportion of yolk and albumen in eggs from Hy-Line Brown laying hens. The inclusion of yellow lupin (LL10-30) did not decrease the proportion (%) of yolk in the egg [28], similar to findings by Laudadio and Tufarelli [30] for the LL18 diet. Drażbo et al. [28] and Rutkowski et al. [7] observed a negative effect *(P* ≤ 0.05) of hen age in birds that were fed a diet with the inclusion of lupin seeds (LAn, LL) on the height of thick albumen and Haugh unit scores. There was also a very significant interaction between the diet and age of hens and these parameters, which is consistent with our findings. According to the classification system by the US Department of Agriculture (2000), eggs with Haugh unit scores of 72 or higher are classified as AA. In our study, the Haugh unit score ranged from 87.45 to 110.3, which indicates high-quality albumen, both in relation to diet and egg production date. Eggs with high-quality albumen (AA) were also obtained in a study by Krawczyk et al. [35]. In our study, there was a correlation between the egg production date and an increased proportion (%) of yolk and a decreased proportion of albumen in the egg. This is consistent with reports by Drażbo et al. [28] for LAn10 and 20 diets and the findings of Rutkowski et al. [7]. The egg production date only had an effect on the weight of yolk in week 5 (55.10 g) and in week 13 (61.4 g).

The significant increase in the saturation of yolk colour is associated with the concentration of pigments in feed. Pigments in the feed supplied to birds are absorbed in the small intestine at a different rate and accumulate in the yolk. The improvement in yolk colour is associated with the persistence of the yellow pigment between fat molecules in the membrane that surrounds the yolk. In our study, the saturation of yolk colour was higher *(P* ≤ 0.05) in eggs from hens that were fed diets with the inclusion of a higher content of narrow-leafed lupin (20% and 25%). This can be explained by the higher concentration of pigments (zeaxanthin, lutein and β-carotene) in lupin seeds [28,42,43,44]. Other studies [8] demonstrated that the lightest yolks (2.01 points) were found in eggs from the control group (Hy-Line Brown), and the colour was correlated with the increased inclusion of yellow lupin in the diet (LL10, 15, 20 and 25). The assessment of colour on the La Roche scale was confirmed by scores in the CIE L*a*b* system. Birds that were fed with soybean meal produced eggs with yolks that were the lightest (L*), less red (a*) and yellower (b*). Similar findings were reported by Lee et al. [31] for a diet with 15% inclusion of whole and dehulled LA seeds. Studies by Dvorák et al. [45] also indicated a positive effect of yellow lupin seeds in the diet of ISA Brown laying hens on the saturation of yolk colour. Experiments by Park et al. [29] demonstrated that the colour of yolks from Hy-Line Brown hens fed diets with 11–22% inclusion of narrow-leafed lupin was comparable to that from the control group (SBM). Hens that were fed a diet with 18% inclusion of white lupin also produced eggs with increased saturation of yolk colour (expressed in points) compared with SBM [30]. The mixture of lupin seeds (LAn and LL) and peas (PS) had no significant effect on the colour of yolk in Hy-Line Brown hens [7]. However, compared with our study in the same breed of hens, the saturation of yolk colour on the La Roche scale was about 5 points higher. This may be attributed to the positive impact of diets that combine different lupin varieties and peas.

According to Rutkowski et al. [8], the higher Haugh unit scores found for eggs from hens that are fed diets containing lupins may be associated with the better elasticity of structural protein, stronger bonds between ovomucin and lysozyme and better properties of egg albumen. Graszkiewicz et al. [46] indicated that the supplementation of a standard feed with vitamins A and E was associated with a higher enzymatic activity of albumen. The researchers pointed to the antioxidant activity of these vitamins in relation to fatty substances involved in metabolic transformations, which result in the presence of oestrogens in the blood serum of birds. In turn, oestrogens are involved in the differentiation of tubal cells that produce albumins and lysozyme. Kopeć et al. [47] indicated that the addition of rapeseed (3.0%) to feed was associated with decreased lysozyme activity in fresh albumen from Tetra SL hens. Experiments by Świerczewska et al. [48] showed the highest activity of lysozyme in albumen in 40-week-old Hy-Line hens *(P* ≤ 0.05). In eggs from Tetra SL laying hens, the highest lysozyme activity in albumen was found in weeks 40 and 50 of egg production [49].

Lupin seeds are a rich source of unsaturated fatty acids (UFAs), which, in turn, increase the level of essential fatty acids in egg yolk [50,51]. Analyses demonstrated that UFAs in white lupin seeds account for about 77%, and saturated fatty acids (SFAs) account for 12.6%. The content of SFA in lupin seeds is lower than that in soybean [52]. Studies by Boshin et al. [50] and Suchỳ et al. [53] revealed that the major PUFAs in lupin seeds were linoleic acid (C18:2n6) and α-linolenic acid (C18:3n3), and they were characterised by a favourable n6/n3 ratio. For this reason, it was assumed in our experiments that the inclusion of blue lupin seed meal in the diet of laying hens could improve the fatty acid composition of the yolk lipid fraction, which was confirmed by the obtained results. The content of linoleic acid in eggs from hens that were fed diets with 20% and 25% inclusion of narrow-leafed lupin was significantly higher (by 1.03% and 0.87%, respectively) compared with the control group (SBM). Moreover, the total content of PUFA and the n6/n3 ratio were also significantly higher in groups that received feed with 20% and 25% inclusion of narrow-leafed lupin seeds. According to Zhang and Kim [54], the consumption of foods containing an increased content of monounsaturated fatty acids (MUFA, including n9 and n6) reduces the level of triglycerides in human blood. Our research on eggs from Hy-Line Brown laying hens found no deteriorating effect of diets containing lupin seed meal on the total MUFA content. Yellow and narrow-leafed lupin in studies by Drażbo et al. [28] and Krawczyk et al. [35] had a significant effect on the composition of fatty acids in the yolk lipid fraction. The intake of meal from narrow-leafed lupin (LA10 and 20) and yellow lupin seeds (LL10, 20 and 30) by Lohmann Brown hens was associated with an increase in the content of pentadecanoic acid (C15:0), heptadecanoic acid (C17:0), linoleic acid (C18:2n6) and the total content of polyunsaturated acids (PUFAs), and the effect was dose-dependent. Lower concentrations were found for palmitic (C16:0) and palmitoleic (C16:1) acids. The results obtained by these authors are consistent with our findings for LA10 and 20 diets. According to Boschin et al. [50] and Suchỳ et al. [53], linoleic acid (C18:2n6) and α-linolenic acid (C18:3n3) have the largest share in the total content of polyunsaturated acids (PUFAs) in lupin seeds, which results in a favourable n6/n3 ratio. This hypothesis was confirmed by our findings for two groups, i.e., D (LAn20) and E (LAn25), with values of 9.15 and 9.02, respectively, and results reported by Krawczyk et al. [35]. The replacement of soybean protein with protein from lupin seeds did not increase the content of saturated fatty acids, either in the present study or in other studies [28,35]. Oleic acid (C18:1n9) is one of the most important fatty acids in the egg yolk. On the other hand, a high content of stearic acid (C18:0) may improve the permeability of the vitellin membrane that encloses the yolk [54]. α-Linolenic acid (n3) and linoleic acid (n3) are not synthesised in the human body or in most animals because of the absence of denaturisers, enzymes that introduce a double bond in the acid molecule next to carbon, and therefore, these fatty acids must be supplied with food. The inclusion of narrow-leafed lupin seeds in the diet of Hy-Line Brown hens was associated with increased content of linoleic acid in the yolk *(P* ≤ 0.05). Omega-3 fatty acids have many beneficial effects on human health; e.g., they reduce the concentration of triacylglycerols in blood plasma, normalise blood pressure and have anticoagulant, antiatherosclerotic and anti-inflammatory and anticancer activity [55]. Shafey [56] reported that the age of laying hens had no effect on the concentration of palmitic (C16:0) or oleic (C18:1n9) acids. However, the content of stearic acid (C18:0) changed over the egg production period and was highest *(P* ≤ 0.05) in eggs from 31-week-old hens, and linoleic acid (C18:2n6) was highest in eggs from 51-week-old hens. The UFA/SFA ratio also changed throughout the egg production period and was highest in eggs laid by 31-week-old birds. The highest C18:1/C18:2 ratio was found for eggs from hens aged 39 weeks. Other studies demonstrated that 1.5–3.0% inclusion of alfalfa seeds in the diet of ISA Brown hens aged 33 and 53 weeks had no significant effect on the total content of saturated fatty acids (SFAs) or unsaturated fatty acids (MUFAs and PUFAs) in yolk lipids. The intake of alfalfa had a positive effect on the n6/n3 ratio compared with the control group [57]. There have been no similar studies in which various levels of narrow-leafed lupin seeds were given to laying hens with a constant level of pea seeds. However, Rutkowski et al. [58] used both these species, as well as yellow lupin, in laying hens’ diets. The authors concluded that the use of 27.68% legume seeds in hens’ diets negatively affected the production, but 19.48% legumes with 8% addition of rapeseed meal could be accepted as an alternative to soybean meal. In our research, pea seeds were used as a constant supplement in feeds (10%). As reported in the other study, pea seeds are characterised by high starch content and slower degradation in the intestines compared with other plants used in poultry feed [59]. A beneficial effect of pea seeds at the 4–16% level in the diets of broiler chickens was found by Dotas et al. [60]. Moreover, McNeill et al. [61] found that 10% content of peas in poultry diets had little effect on the production, but 20% peas reduced the feed intake of birds. Among the sugar group, only starch, whose content in the seeds of the pea var. Muza is over 44%, is digested by endogenous alpha-amylase and effectively used in the digestive system of birds [62]. Therefore, it can be concluded that the addition of peas has no negative effect and only enriches the diet for poultry.

## 5. Conclusions

The use of 10–20% lupin seeds with pea seeds at the 10% level in laying hens’ diets had no negative effect on the major traits of eggs, including weight and morphological composition, or on the content of lysozyme and its activity. Too much lupin seed content in feed (25%) is not recommended because of the effect on the weight of eggs. Our experiment revealed that the inclusion of protein-rich seeds also had a positive effect on the ratio of omega fatty acids, especially with 10–20% content of lupin seeds in laying hens’ diets. Examination of the colour of the yolk revealed that increasing the content of lupin seeds in feed had a stronger colour effect, which is a trait that is in line with consumers’ requirements. The use of narrow-leafed lupin and pea seeds could be proposed for laying hens in countries where the environmental conditions do not allow for the production of soybean crops. These results suggest the potential for a wider range of choices in the consumer market. Nowadays, not everybody wants to buy products from animals that were raised on GMO feed. Our results provide possible alternative protein sources in poultry diets.

## Figures and Tables

**Table 1:** Composition and nutritional value of feeds for laying hens.

Components (%)	Treatment ^1^				
	A	B	C	D	E
Wheat	59.603	52.033	48.100	45.920	44.964
Narrow-leafed lupin	-	10.000	15.000	20.000	25.000
Soybean meal (SBM)	22.163	9.600	8.000	5.000	-
Limestone < 2 mm	4.000	4.000	4.000	4.000	4.000
Limestone > 2 mm	5.057	5.000	5.000	5.000	5.000
Pea	-	10.000	10.000	10.000	10.000
Rapeseed oil	6.186	6.217	6.814	7.000	7.800
Monocalcium phosphate	1.682	1.702	1.690	1.700	1.700
NaCl	0.200	0.181	0.200	0.2000	0.189
DL-Methionine	0.222	0.200	0.200	0.2000	0.220
NaCHO_3_	0.280	0.300	0.290	0.290	0.290
Lysine	0.025	0.100	0.900	0.090	0.170
Threonine	0.006	0.060	0.023	0.020	0.050
Premix ^2^	0.500	0.500	0.500	0.500	0.500
Valine	0.077	0.077	0.060	0.050	0.070
Tryptophan	-	0.020	0.023	0.025	0.040
Calculated nutritional value of feed					
Metabolisable energy (MJ/kg)	11.30	11.30	11.30	11.30	11.30
Metabolisable energy (kcal) (%)	2699.00	2699.00	2699.00	2699.00	2699.00
Crude protein	16.20	16.20	16.20	16.20	16.20
Calcium	3.50	3.50	3.50	3.50	3.50
*P*-available	0.39	0.39	0.39	0.39	0.39
Lysine	0.75	0.75	0.75	0.75	0.75
Methionine + Cystine	0.63	0.63	0.63	0.63	0.63
Tyrosine	0.16	0.16	0.16	0.16	0.16
Threonine	0.53	0.53	0.53	0.53	0.53
Valine	0.68	0.68	0.68	0.68	0.68

^1^ TREATMENT: A, control with soybean meal; B, 10% lupin seeds; C, 15% lupin seeds; D, 20% lupin seeds; E, 25% lupin seeds; ^2^ The vitamin and mineral premix provides per kg of diet: Cu, 10 mg; Fe, 60 mg; Mn, 80 mg; Zn, 60 mg; I, 1.5 mg; Se, 0.3 mg; vitamin A, 10.000 IU; vitamin D, 2500 IU; vitamin E, 25 IU; vitamin K, 1.0 mg; vitamin B1, 2.0 mg; vitamin B2, 8.0 mg; vitamin B6, 2.5 mg; vitamin B12, 0.01 mg; vitamin PP (nicotinamide pancreatic polypeptide), 30.0 mg; vitamin B5, 15.0 mg; vitamin B9, 0.5 mg; and biotin, 0.15 mg.

**Table 2:** Chemical composition of narrow-leafed lupin seeds (cv. Boruta) and pea seeds (cv. Muza).

Parameter ^1^	Narrow-Leafed Lupin, cv. Boruta	Pea Seeds, cv. Muza
Dry matter %	88.62	86.65
Crude ash %	3.78	3.14
Crude protein %	36.88	27.57
Crude fibre %	15.09	6.34
ADF ^1^ %	21.43	7.97
NDF ^2^ %	25.92	13.88
Crude fat %	5.81	1.32
Starch %	-	44.23
Metabolisable energy (MJ/kg)	20.73	19.45
Metabolisable energy (kcal/kg)	4951.28	4645.55
Viscose, cP	1.21	1.29
Amino acids, %		
Aspartic acid %	8.91	10.49
Threonine %	3.15	3.54
Serine %	4.11	4.38
Glutamic acid %	23.77	19.46
Proline %	6.52	5.77
Glycine %	4.01	3.83
Alanine %	3.33	3.81
Valine %	3.72	4.35
Isoleucine %	3.68	3.66
Leucine %	6.64	6.63
Tyrosine %	3.07	3.26
Phenylalanine %	3.46	5.00
Histidine %	2.91	3.37
Lysine %	4.49	6.52
Arginine %	11.65	8.82
Total amino acids %:	39.39	42.53
Minerals, g/kg DM		
Calcium g/kg DM	3.33	1.27
Potassium g/kg DM	13.45	12.72
Phosphorus g/kg DM	6.84	5.10
Sodium g/kg DM	0.08	0.062
Magnesium g/kg DM	2.10	1.47
Manganese g/kg DM	0.13	0.02
Copper g/kg DM	0.04	0.02
Iron g/kg DM	0.07	0.07
Zinc g/kg DM	0.07	0.06
Alkaloid profile, %		
Total alkaloids (mg/kg)	440	-
Angustifoline %	12.45	-
Isolupanine %	4.56	-
Lupanine %	56.17	-
130H Lupanine %	26.72	-
Sparteine %	-	-
Lupinine %	-	-
Oligosaccharides, g/kg DM		
Oligosaccharides, g/kg DM	8.77	8.34
Raffinose g/kg DM	1.20	0.90
Stachyose g/kg DM	5.61	3.86
Verbascose g/kg DM	1.96	3.59
P-phytate (g)	0.42	0.44

^1^ADF, acid detergent fibre; ^2^NDF, neutral detergent fibre.

**Table 3:** Egg weight, egg shape index and egg surface area (means ± SD *).

Parameter		Egg Weight (g)	Egg Shape Index (%)	Egg Surface Area (cm^2^)
Treatment ^1^	A	63.27 ^a,b^	77.32	75.22 ^b^
	B	63.79 ^a^	77.37	75.64 ^a^
	C	62.78 ^b^	77.48	74.85 ^b,c^
	D	63.03 ^a,b^	77.50	75.02 ^b,c^
	E	61.27 ^c^	77.93	73.65 ^c^
	± SD	± 6.72	± 3.14	± 4.38
	*P*-value	0.000	0.147	0.000
Egg production period (2.5-week intervals)	I	53.52 ^g^	78.82 ^a^	67.36 ^h^
	II	58.51 ^f^	78.85 ^a^	71.45 ^g^
	III	61.23 ^e^	78.88 ^a^	73.62 ^f^
	IV	63.14 ^c,d^	78.64 ^a,b^	75.15 ^d,e^
	V	64.50 ^a–c^	77.72 ^a,b^	76.20 ^a–d^
	VI	64.79 ^a–c^	78.18 ^a,b^	76.44 ^a–d^
	VII	62.22 ^d,e^	77.61 ^a,b^	74.44 ^e,f^
	VIII	63.50 ^b–d^	78.64 ^a,b^	75.41 ^c–e^
	IX	66.28 ^a^	76.90 ^c,d^	77.65 ^a^
	X	64.33 ^a–c^	76.32 ^d,e^	76.09 ^b–d^
	XI	65.09 ^a,b^	75.30 ^d,e^	76.68 ^a–c^
	XII	65.37 ^a^	76.41 ^d,e^	76.89 ^a,b^
	XIII	65.78 ^a^	76.04 ^d,e^	77.22 ^a,b^
	± SD	± 4.47	± 2.96	± 3.51
	*P*-value	0.000	0 000	0.000
	Interaction	0.618	0.000 ^x^	0.663

Different letters (a, b, c, d, e, f, g, h) indicate a significant difference between treatments (A–E) and periods (I–XIII) at *P* < 0.05; ^1^ TREATMENT: A, control with soybean meal; B, 10% lupin seeds; C, 15% lupin seeds; D, 20% lupin seeds; E, 25% lupin seeds. * SD, standard deviation; ^x^ interaction between factors.

**Table 4:** Eggshell parameters (means ± SD *).

Parameter		Shell Strength (kg/cm^2^)	Shell Weight (g)	Shell Proportion in the Egg (%)	Shell Thickness (mm)
Treatment ^1^	A	4.21	6.31 ^a^	9.99 ^a^	0.359
	B	4.13	6.14 ^b,c^	9.64 ^c^	0.355
	C	4.11	6.04 ^c^	9.65 ^c^	0.354
	D	4.19	6.16 ^b^	9.80 ^b,c^	0.352
	E	4.05	6.04 ^c^	9.87 ^a,b^	0.350
	± SD	± 0.89	± 0.69	± 0.95	± 0.06
	*P*-value	0.128	0.000	0.000	0.601
Egg production period (2.5-week intervals)	I	4.12 ^c,d^	5.25 ^f^	9.83 ^b,c^	0.334 ^b^
	II	4.02 ^d,e^	5.58 ^e^	9.54 ^c–e^	0.337 ^a,b^
	III	4.55 ^a–c^	5.94 ^d^	9.72 ^b–d^	0.352 ^a,b^
	IV	4.61 ^a^	6.02 ^c^	9.56 ^c–e^	0.360 ^a,b^
	V	4.44 ^a–c^	6.03 ^c^	9.39 ^de^	0.360 ^a,b^
	VI	3.99 ^d,e^	6.05 ^c^	9.22 ^e^	0.362 ^a,b^
	VII	3.98 ^d,e^	6.08 ^c^	9.79 ^b,c^	0.349 ^a,b^
	VIII	3.98 ^d,e^	6.13 ^c^	9.70 ^b–d^	0.348 ^a,b^
	IX	4.02 ^d,e^	6.37 ^b^	9.63 ^b–d^	0.355 ^a,b^
	X	4.07 ^d,e^	6.39 ^b^	9.97 ^a,b^	0.364 ^a,b^
	XI	3.93 ^d,e^	6.66 ^a^	10.25 ^a^	0.367 ^a^
	XII	4.00 ^d,e^	6.66 ^a^	10.22 ^a^	0.356 ^a,b^
	XIII	4.06 ^d,e^	6.70 ^a^	10.22 ^a^	0.362 ^a,b^
	± SD	± 0.85	± 0.57	± 0.91	± 0.05
	*p*-value	0.000	0.000	0.000	0.006
	Interaction	0.037 ^x^	0.000 ^x^	0.000 ^x^	0.213

Different letters (a, b, c, d, e, f) indicate a significant difference between treatments (A–E) and periods (I–XIII) at *P* < 0.05; ^1^ TREATMENT: A, control with soybean meal; B, 10% lupin seeds; C, 15% lupin seeds; D; 20% lupin seeds; E, 25% of lupin seeds. * SD, standard deviation; ^x^ interaction between factors

**Table 5:** Characteristics of egg components (means ± SD *).

Parameter		Weight (g)				Proportion in Egg (%)				Height of Thick Albumen (mm)	Haugh Units (HU)
		of Thick Albumen	of Thin Albumen	Total Albumen	Yolk	Thick Albumen	Thin Albumen	Total Albumen	Yolk		
Treatment ^1^	A	22.12 ^a,b^	20.51 ^b^	42.56 ^b^	14.44 ^a^	34.84 ^b,c^	32.42 ^b^	67.26 ^c^	22.74 ^a,b^	8.80	91.92
	B	21.95 ^a,b^	21.35 ^a^	43.33 ^a^	14.27 ^a,b^	34.46 ^c^	33.45 ^a^	67.92 ^a^	22.46 ^c^	8.94	93.24
	C	22.23 ^b^	20.30 ^b,c^	42.53 ^b^	14.22 ^a,b^	35.38 ^b^	32.35 ^b,c^	67.73 ^b^	22.62 ^a,b^	8.97	93.56
	D	22.93 ^a^	19.75 ^c^	42.66 ^b^	14.19 ^a,b^	36.35 ^a^	31.35 ^c^	67.71 ^b,c^	22.50 ^b^	8.83	92.88
	E	21.42 ^c^	19.75 ^c^	41.17 ^b^	14.07 ^b^	34.93 ^b,c^	32.27 ^c^	67.21 ^b,c^	22.92 ^a^	8.75	93.08
	± SD	3.40	3.86	5.42	2.01	4.47	2.84	2.84	2.50	1.71	9.79
	*p*-value	0.000	0.000	0.000	0.021	0.000	0.000	0.001	0.001	0.492	0.439
Egg production period (2.5-week intervals)	I	19.12 ^f^	18.64 ^e^	37.75 ^f^	10.48 ^h^	35.70 ^a,b^	34.78 ^b^	70.49 ^a^	19.69 ^g^	9.72 ^a^	100.30 ^a^
	II	19.44 ^f^	21.67 ^a^	41.11 ^e^	11.82 ^g^	33.23 ^e^	36.96 ^a^	70.19 ^a^	20.27 ^g^	9.93 ^a^	99.42 ^a^
	III	20.95 ^d,e^	21.19 ^a,b^	41.14 ^d,e^	13.12 ^f^	34.21 ^c^	34.61 ^b^	68.81 ^b^	21.47 ^f^	9.22 ^a,b^	96.04 ^b^
	IV	21.51 ^d,e^	21.85 ^a^	43.12 ^b,c^	13.80 ^e^	34.11 ^c^	34.58 ^b^	68.38 ^b,c^	21.75 ^e,f^	9.27 ^a,b^	95.90 ^b^
	V	22.67 ^d,e^	21.75 ^a^	44.40 ^a,b^	14.04 ^e^	35.17 ^b,c^	33.62 ^b,c^	68.79 ^b,c^	21.83 ^e,f^	9.32 ^a,b^	95.85 ^b^
	VI	21.97 ^d,e^	21.62 ^a^	43.57 ^a–c^	15.27 ^c^	33.82 ^c,d^	33.36 ^b–d^	67.19 ^d,e^	23.60 ^b,c^	9.35 ^a,b^	94.66 ^b,c^
	VII	21.93 ^d,e^	19.65 ^c–e^	41.58 ^d,e^	14.56 ^d^	35.19 ^b,c^	31.58 ^d–f^	66.78 ^e,f^	23.44 ^c,d^	8.85 ^b,c^	93.09 ^c^
	VIII	22.15 ^c,d^	20.22 ^b,c^	42.29 ^c–e^	15.07 ^c^	34.75 ^c^	31.77 ^d–f^	66.51 ^e,f^	23.78 ^a–c^	8.69 ^b,c^	91.87 ^c,d^
	IX	23.42 ^a,b^	21.59 ^a,b^	45.02 ^a^	14.94 ^c,d^	35.33 ^a,b^	32.47 ^c,e^	67.79 ^c,d^	22.57 ^d,e^	9.02 ^b^	93.59 ^c^
	X	22.94 ^b^	19.70 ^a–c^	42.64 ^c–e^	15.31 ^b,c^	35.67 ^a,b^	30.52 ^f,g^	66.18 ^f,g^	23.85 ^a–c^	8.33 ^c,d^	90.31 ^c,d^
	XI	23.36 ^a,b^	19.64 ^c–e^	43.02 ^b–d^	15.42 ^a,b^	35.94 ^a,b^	30.10 ^f,g^	66.04 ^f,g^	23.74 ^a–c^	8.28 ^c,d^	89.57 ^e^
	XII	24.28 ^a^	18.54 ^e^	42.82 ^c,d^	15.89 ^a^	37.26 ^a^	28.11 ^h^	65.37 ^g^	24.41 ^a^	8.20 ^d^	89.29 ^e^
	XIII	23.79 ^a^	19.27 ^c–e^	43.09 ^b–d^	16.00 ^a^	36.21 ^a^	29.22 ^g,h^	65.43 ^g^	24.35 ^a^	7.99 ^e^	87.45 ^f^
	± SD	3.09	3.68	3.88	1.23	4.26	2.32	2.32	1.98	1.61	9.02
	*p*-value	0.000	0.000	0.000	0.000	0.000	0.000	0.000	0.000	0.000	0.000
	Interaction	0.000 ^x^	0.000 ^x^	0.000 ^x^	0.348	0.000 ^x^	0.000 ^x^	0.001 ^x^	0.297	0.000 ^x^	0.000 ^x^

Different letters (a, b, c, d, e, f, g, h) indicate a significant difference between treatments (A–E) and periods (I–XIII) at *P* < 0.05; ^1^ GROUP: A, control with soybean meal; B, 10% of lupin seeds; C, 15% of lupin seeds; D, 20% of lupin seeds; E, 25% of lupin seeds. * SD, standard deviation; ^x^ interaction between factors.

**Table 6:** Yolk colour (means ± SD *).

Parameter		La Roche (points)	Colour		
			L*	a*	b*
Treatment ^1^	A	6.29 ^d^	48.64 ^a^	− 1.72 ^c^	17.65 ^c^
	B	7.51 ^c^	48.39 ^a^	− 1.20 ^b^	21.82 ^b^
	C	8.01 ^b^	47.63 ^b^	− 0.04 ^a^	23.56 ^a^
	D	8.40 ^a^	47.23 ^b^	0.25 ^a^	23.60 ^a^
	E	8.54 ^a^	47.28 ^b^	0.31 ^a^	23.51 ^a^
	± SD	4.09	4.95	5.49	6.19
	*P*-value	0.000	0.000	0.000	0.000
Egg production period (2.5-week intervals)	I	11.37 ^c^	46.33 ^c^	4.62 ^b^	20.97 ^d^
	II	12.33 ^b^	42.90 ^e^	5.28 ^a,b^	19.36 ^e–g^
	III	12.94 ^a^	44.69 ^d^	5.21 ^ab^	17.28 ^h^
	IV	12.89 ^a^	43.65 ^d,e^	5.28 ^ab^	17.21 ^h^
	V	12.59 ^a,b^	43.13 ^e^	5.80 ^a^	18.37 ^g,h^
	VI	4.27 ^f^	48.44 ^b^	0.72 ^c^	20.52 ^d,e^
	VII	4.54 ^e,f^	47.22 ^c^	− 4.17 ^d^	20.05 ^d–f^
	VIII	4.14 ^f^	48.53 ^b^	− 4.51 ^d^	18.80 ^f,g^
	IX	4.17 ^f^	47.59 ^b,c^	− 4.87 ^d^	19.32 ^e–g^
	X	4.34 ^f^	48.82 ^b^	− 4.74 ^d^	19.13 ^f,g^
	XI	4.60 ^e,f^	54.39 ^a^	− 5.97 ^e^	29.46 ^c^
	XII	4.91 ^d,e^	53.92 ^a^	− 5.92 ^e^	31.03 ^b^
	XIII	5.27 ^d^	53.97 ^a^	− 6.11 ^e^	33.72 ^a^
	± SD	1.44	3.06	1.25	5.09
	*P*-value	0.000	0.000	0.000	0.000
	Interaction	0.000 ^x^	0.001 ^x^	0.000 ^x^	0.000 ^x^

Different letters (a, b, c, d, e, f, g, h) indicate a significant difference between treatments (A–E) and periods (I–XIII) at *P* < 0.05; ^1^ TREATMENT: A, control with soybean meal; B, 10% lupin seeds; C, 15% lupin seeds; D; 20% lupin seeds; E, 25% lupin seeds. * SD, standard deviation; ^x^ interaction between factors.

**Table 7:** Concentration and activity of lysozyme in albumen (means, V *).

Parameter			Concentration of Lysozyme (%) in Albumen		Lysozyme Activity (U/mg) in Albumen	
			Thick	Thin	Thick	Thin
Treatment ^1^		A	0.210 ^a^	0.429 ^b,c^	44,569 ^a^	9144 ^b^
		B	0.190 ^b^	0.424 ^c^	40,577 ^b^	90,190 ^b^
		C	0.202 ^a,b^	0.422 ^c^	42,956 ^a,b^	89,765 ^b^
		D	0.200 ^a,b^	0.441 ^a,b^	42,477 ^a,b^	88,263 ^b^
		E	0.200 ^a,b^	0.452 ^a^	42,385 ^a,b^	96,165 ^a^
		V (%)	20.25	11.24	20.16	13.11
		*P*-value	0.004	0.000	0.005	0.000
Egg production period (2.5-week intervals)		II	0.200 ^b–d^	0.424 ^c^	42,803 ^b–d^	75,399 ^e^
		III	0.188 ^c,d^	0.486 ^a^	39,882 ^c,d^	103,313 ^a^
		IV	0.214 ^a^	0.448 ^b–d^	45,574 ^a^	95,228 ^c,d^
		V	0.207 ^b–d^	0.444 ^b–d^	43,982 ^b–d^	94,444 ^c–e^
		VI	0.208 ^b–d^	0.430 ^b–d^	44,152 ^b–d^	91,389 ^c–e^
		VII	0.210 ^b–d^	0.449 ^b,c^	44,353 ^b–d^	95,459 ^b^
		VIII	0.194 ^b–d^	0.429 ^c^	41,186 ^b–d^	91,217 ^d^
		IX	0.162 ^d^	0.406 ^d^	34,616 ^d^	86,371 ^e^
		X	0.210 ^b–d^	0.433 ^b–d^	44,555 ^b–d^	92,000 ^c–e^
		XI	0.198 ^b–d^	0.404 ^d^	40,275 ^b–d^	86,145 ^e^
		XII	0.210 ^b–d^	0.433 ^b–d^	44,781 ^b,c^	92,297 ^c–e^
		XIII	0.212 ^b^	0.416 ^d^	45,003 ^b^	87,780 ^d^
		V (%)	19.27	10.27	19.20	11.58
		*P*-value	0.000	0.000	0.000	0.000
		Interaction	0.001 ^x^	0.000 ^x^	0.000 ^x^	0.000 ^x^

Different letters (a, b, c, d, e) indicate a significant difference between treatments (A–E) and periods (I–XIII) at *P* < 0.05; ^1^ TREATMENT: A, control with soybean meal; B, 10% lupin seeds; C, 15% lupin seeds; D; 20% lupin seeds; E, 25% lupin seeds. * V, coefficient of variation; ^x^ interaction between factors.

**Table 8:** Content of fatty acids (%) in egg yolk lipids (means ± SD *).

Fatty acids	Treatment ^1^					± SD	Egg Production Period			*P*-Value		
	A	B	C	D	E		I	II	III	Group	Egg Production Date	Interaction
C14:0	0.34 ^a^	0.33 ^a^	0.32 ^b^	0.28 ^b^	0.30 ^b^	0.04	0.29 ^b^	0.33 ^a^	0.33 ^a^	0.000	0.000	0.004 ^x^
C15:0	0.11 ^b^	0.12 ^a^	0.12 ^a^	0.12 ^a^	0.12 ^a^	0.01	0.12 ^a^	0.12 ^a^	0.11 ^b^	0.001	0.002	0.767
C16:0	41.81 ^a^	41.56 ^a^	40.20 ^b^	40.15 ^b^	40.06 ^b^	1.28	40.61	41.03	40.60	0.000	0.237	0.011 ^x^
C16:1	1.19 ^a^	1.00 ^b^	0.93 ^b,c^	0.79 ^c^	0.80 ^c^	0.14	0.99 ^a^	0.86 ^b^	0.96 ^b^	0.000	0.007	0.120
C17:0	0.24 ^c^	0.28 ^b, c^	0.29 ^b^	0.30 ^a^	0.30 ^a^	0.02	0.29 ^a^	0.29 ^a^	0.27 ^b^	0.000	0.020	0.819
C18:0	14.90	14.72	15.07	15.97	15.98	1.42	15.64	15.02	15.33	0.125	0.305	0.013 ^x^
C18:1n9	28.53	28.61	29.41	28.30	28.57	1.59	28.45	28.72	28.89	0.237	0.537	0.000 ^x^
C18:2n6	9.91 ^c^	10.36 ^b,c^	10.62 ^b,c^	10.94 ^a^	10.78 ^b^	0.42	10.52	10.53	10.54	0.000	0.962	0.118
C18:3n3	1.20	1.27	1.30	1.24	1.22	0.15	1.11 ^b^	1.31 ^a^	1.32 ^a^	0.141	0.000	0.033 ^x^
C20:1n9	0.15	0.15	0.15	0.15	0.15	0.01	0.15 ^a^	0.14 ^b^	0.15 ^a^	0.548	0.000	0.267
C20:2n6	0.10	0.09	0.09	0.10	0.10	0.01	0.10	0.10	0.09	0.519	0.344	0.009 ^x^
C22:0	1.16	1.12	1.13	1.27	1.23	0.20	1.33 ^a^	1.14 ^a^	1.07 ^b^	0.140	0.000	0.013 ^x^
C24:0	0.36	0.38	0.38	0.40	0.38	0.06	0.39 ^b^	0.40 ^a^	0.35 ^a,b^	0.739	0.008	0.120

Different letters (a, b, c) indicate a significantly difference between treatments (A–E) and periods (I–XIII) at *P* < 0.05; ^1^ TREATMENT: A, control with soybean meal; B, 10% lupin seeds; C, 15% lupin seeds; D, 20% lupin seeds; E, 25% lupin seeds; saturated fatty acids (SFA), C14:0 + C15:0 + C16:0 + C17:0 + C18:0 + C22:0 + C24:0; monounsaturated fatty acids (MUFA), C16:1 + C18:1n9 + C20:1n9; polyunsaturated fatty acids (PUFA), C18:2n6 + C18:3n3 + C20:2n6. * SD, standard deviation; ^x^interaction between factors.

## References

[1] Hammershøj M., Steenfeldt S. (2005). Effects of blue lupin (Lupinus angustifolius) in organic layer diets and supplementation with foraging material on egg production and some egg quality parameters. Poult. Sci..

[2] Hejdysz M., Rutkowski A. (2015). Aktualne problemy żywienia zwierząt monogastrycznych–podaż pasz wysokobiałkowych i białkowe bezpieczeństwo kraju. Przeg. Hodow.

[3] Kaczmarek S.A., Hejdysz M., Kubis M., Kasprowicz-Potocka M., Rutkowski A. (2016). The nutritional value of yellow lupin (Lupinus luteus L.) for broilers. Anim. Feed Sci. Technol..

[4] Kubiś M., Kaczmarek S.A., Nowaczewski S., Adamski M., Hejdysz M., Rutkowski A. (2018). Influence of graded inclusion of white lupin (*Lupinus albus*) meal on performance, nutrient digestibility and ileal viscosity of laying hens. Brit. Poult. Sci..

[5] Nalle C.L., Ravindran V., Ravindran G. (2011). Nutritional value of narrow-leafed lupin (*Lupinus angustifolius*) for broilers. Brit. Poult. Sci..

[6] Kaczmarek S.A., Kasprowicz-Potocka M., Hejdysz M., Mikuła R., Rutkowski A. (2014). The nutritional value of narrow-leafed lupin (Lupinus angustifolius) for broilers. J. Anim. Feed Sci..

[7] Rutkowski A., Kaczmarek S.A., Hejdysz M., Nowaczewski S., Jamroz D. (2015). Concentrates made from legume seeds (*Lupinus angustifolius*, *Lupinus luteus* and *Pisum sativum*) and rapeseed meal as protein sources in laying hen diets. Ann. Anim. Sci..

[8] Rutkowski A., Hejdysz M., Kaczmarek S., Adamski M., Nowaczewski S., Jamroz D. (2017). The effect of addition of yellow lupin seeds (*Lupinus luteus* L.) to laying hen diets on performance and egg quality parameters. J. Anim. Feed Sci..

[9] Roberts J.R. (2004). Factors Affecting Egg Internal Quality and Egg Shell Quality in Laying Hens. J. Poult. Sci..

[10] Eisen E.J., Bohren B.B., McKean H.E. (1962). The Haugh Unit as a Measure of Egg Albumen Quality. Poult. Sci..

[11] Williams I.O., Agiog M.A., Ekpe O.O., Aletan U.I., Edet E.O., Atangwho I.J. (2009). Nutritional quality of frist generation QPM diet and its effect on some biological indices of Albino Wistar Rats. Am. J. Food Technol..

[12] Banaszewska D., Biesiada-Drzazga B., Ostrowski D., Drabik K., Batkowska J. (2019). The impact of breeder age on egg quality and lysozyme activity. Tur. J. Vet. Anim. Sci..

[13] Adamski M., Kucharska-Gaca J., Kuźniacka J., Gornowicz E., Lewko L., Kowalska E. (2016). Effect of goose age on morphological composition of eggs and on level and activity of lysozyme in thick albumen and amniotic fluid. Europ. Poult. Sci..

[14] Banaszak M., Kuźniacka J., Biesek J., Maiorano G., Adamski M. (2020). Meat quality of traits and fatty acid composition of breast muscles from ducks fed with yellow lupin. Animals.

[15] Paganelli C.V., Olszowka A., Ar A. (1974). The avian egg: Surface area, volume, and density. Condor.

[16] Williams K.C. (1992). Some factors affecting albumen quality with particular reference to Haugh units score. World’s Poult. Sci. J..

[17] Leśnierowski G., Kijowski J. (1995). Methods for testing enzymatic activity and quantification of lysozyme from chicken egg protein. Przem. Spoż..

[18] Folch J., Less M., Stanley G.H.S. (1957). A simple method for the isolation and purification of total lipids from animal tissue. J. Biol. Chem..

[19] AOAC (2007). Official Methods of Analysis of the Association Official Analytical Chemists.

[20] Kuhla S., Ebmeier C. (1981). Untersuchungen zum Tanningehalt in Ackerbohnen. Arch. Tierernährung..

[21] Haug W., Lantzsch H.J. (1983). Sensitive method for the rapid determination of phytate in cereals and cereal products. J. Sci. Food Agri..

[22] Johnston S.A., Gous R.M. (2007). Modeling the changes in the proportions of the egg components during a laying cycle. Brit. Poult. Sci..

[23] Rizzi C., Chiericato G.M. (2005). Organic farming production. Effect of age on the productive yield and egg quality of hens of two commercial hybrid lines and two local breeds. Ital. J. Anim. Sci..

[24] Van den Brand H., Parmentier H.K., Kemp B. (2004). Effects of housing system (outdoor vs. cages) and age of laying hen on egg characteristics. Brit. Poult. Sci..

[25] Zemková L., Simeonovová J., Lichovníková M., Somerlíková K. (2007). The effects of housing system and age of hens on the weight and cholesterol concentration of the egg. Czech J. Anim. Sci..

[26] Suk Y.O., Park C. (2001). Effect of breed and age of hens on the yolk to albumen ratio in two different genetics stocks. Poult. Sci..

[27] Silversides F.G., Scott T.A. (2001). Effect of storage and layer age on quality of eggs from two lines of hens. Poult. Sci..

[28] Drażbo A., Mikulski D., Zduńczyk Z., Szmatowicz B., Rutkowski A., Jankowski J. (2014). Fatty acid composition, physicochemical and sensory properties of eggs from laying hens fed diets containing blue lupin seeds. Europ. Poult. Sci..

[29] Park J.H., Lee S.I., Kim I.H. (2016). Effects of lupin seed supplementation on egg production performance, and qualitative egg traits in laying hens. Vet. Med..

[30] Laudadio V., Tufarelli V. (2011). Influence of substituting dietary soybean meal for dehulled-micronized lupin (*Lupinus albus* cv. Multitalia) on early phase laying hens production and egg quality. Livestock Sci..

[31] Lee M.R.F., Parkinson S., Fleming H.R., Theobald V.J., Leemans D.K., Burgess T. (2016). The potential of blue lupins as protein source in the diets of laying hens. Vet. Anim. Sci..

[32] Koivunen E., Tuunainen P., Valkonen E., Rossow L., Valaja J. (2014). Use of faba beans (*Vicia faba* L.) in diets of laying hens. Agric. Food Sci..

[33] Fru-Nij F., Niess E., Pfeffer E. (2007). Effect of graded replacement of soyabean meal by faba beans (*Vicia faba* L.) or field peas (*Pisum sativum* L.) in rations for laying hens on egg production and quality. J. Poult. Sci..

[34] Abas K.A., Al-Sardary S.Y. (2007). Effects of faba beans inclusion into hen’s diet and age influence on egg-shell quality. Slovak J. Anim. Sci..

[35] Krawczyk M., Przywitowski M., Mikulski D. (2015). Effect of yellow lupin (*L. luteus*) on the egg yolk fatty acid profile, the physicochemical and sensory properties of eggs, and laying hen performance. Poult. Sci..

[36] Laudadio V., Ceci E., Lastella N.M.B., Introna M., Tufarelli V. (2014). Low-fiber alfalfa (*Medicago sativa* L.) meal in the laying hen diet: Effects on productive traits and egg quality. Poult. Sci..

[37] Beyene G., Ameha N., Urge M., Estifanos A. (2014). Replacing soybean meal with processed Lupin (Lupinus Albus) meal as poultry layers feed. Livestock Res Rural Dev..

[38] Rodrigez-Navarro A.O., Kalin A.O., Nys Y., Garcia-Ruiz J.M. (2002). Influence of the microstructure on the shell strength of eggs laid by hens of different ages. Brit. Poult. Sci..

[39] Mitsuoka T. (1990). Bifidobacteria and their role in human health. J. Ind. Microbiol..

[40] Martinez-Villaluenga C., Frias J., Vidal-Velverde C. (2006). Functional lupin seeds (Lupinus albus L. and Lupinus luteus L.) after extraction of α-galactosides. Food Chem..

[41] Ketta M., Tumova E. (2018). Eggshell characteristics and cuticule deposition in three laying hen genotypes housed in enriched cages and on litter. Czech J. Anim. Sci..

[42] Franchini A. (2007). Egg quality traits of laying hens reared in organic and conventional system. Ital. J. Anim. Sci..

[43] Mansoub N.H. (2011). Effect of different level of Lucerne extract on performance, egg quality and some blood parameters of laying hens. Ann. Biol. Res..

[44] Wang S., Errington S., Yap H.H. Studies on carotenoids from lupin seeds. Proceedings of the Lupins for Health and Wealth’Proceedings of the 12th International Lupin Conference 2008.

[45] Dvořák P., Straková E., Kunowá J., Kunowá V. (2007). Egg yolk color depends upon the composition of the feeding mixture for laying hens. Acta Vet. Brno.

[46] Graszkiewicz A., Kaźmierska M., Niedbalska J. (2007). Effect of the addition of mineral-humine preparations and antioxidants to laying feed on the activity of lysozyme and cystatin in egg white. Żywn. Nauk. Technol. Ja..

[47] Kopeć W., Skiba T., Korzeniowska M., Bobak Ł., Trziszka T. (2005). Activity of protease inhibitors and lysozyme of hen’s egg white depending on feed modification and egg storage. Pol. J. Food. Nutr. Sci..

[48] Świerczewska E., Skiba T., Sokołowska A., Noworyta-Głowacka J., Kopeć W., Koeniowska M., Bobak L. Egg white biologically active proteins activity in relation to laying hen’s age. Proceedings of the XIth European Symposium on the Quality of Eggs and Egg Products Doorwerth.

[49] Trziszka T., Saleh Y., Kopeć W., Wojciechowksa-Smardz I., Oziemblowski M. (2004). Changes in the activity of lysozyme and cystatin depending on the age of layers and egg treatment during processing. Arch. Geflügelk..

[50] Boschin G., D’Agostina A., Annicchiarico P., Arnoldi A. (2008). Effect of genotype and environment on fatty acid composition of Lupinus albus L. seed. Food Chem..

[51] Resta D., Boschin G., D’Agostina A., Arnoldi A. (2008). Evaluation of total quinolizidine alkaloids content in lupin flours, lupin-based ingredients and foods. Mol. Nutr. Food Res..

[52] Uzun B., Arslan C., Karhan M., Toker C. (2007). Fat and fatty acids of white lupin (*Lupinus albus* L.) in comparison to sesame (*Sesamum indicum* L.). Food Chem..

[53] Suchỳ P., Straková E., Kroupa L., Večerek V. The fatty acid content of oil from seeds of some lupin varieties. Proceedings of the 12th International Lupin Conference ‘Lupins for Health and Wealth’.

[54] Zhang Z.F., Kim I.H. (2014). Effects of dietary olive oil on egg quality, serum, cholesterol characteristics, and yolk fatty acid concentrations in lying hens. J. Appl. Anim. Res..

[55] Marciniak-Łukasiak K. (2011). The role and importance of OMEGA-3 fatty acids. Żywn.-Nauk. Technol. Ja..

[56] Shafey T.M. (1996). The relationship between age and egg production egg components and lipoprotein, lipids and fatty acids of the plasma and eggs of laying hens. J. Appl. Anim. Res..

[57] Grela E.R., Ognik K., Czech A., Matras J. (2014). Quality assessment of eggs from laying hens fed mixture with Lucerne protein concentrate. J. Anim. Feed Sci..

[58] Rutkowski A., Kaczmarek S., Nowaczewski S., Jamroz D. (2014). Concentrates From the sugar group, only starch, whose content in the seeds of the pea variety Muza is over 44%, is digested by endogenous alpha-amylase and effectively used in the digestive system of birds [Wiseman, 2006]. Therefore, it can be concluded that the addition of peas has no negative effect, but only supplements the diet for poultry.Made from: Legume Seeds (*Lupinus Angustifolius*, *Lupinus Luteus* and *Pisum Sativum*) and Rapeseed Meal as Protein Sources in Laying Hen Diets. Ann. Anim. Sci..

[59] Kuźniacka J., Biesek J., Banaszak J., Rutkowski A., Kaczmarek S., Adamski M., Hejdysz M. (2020). Effect of Dietary Protein Sources Substituting Soybean Meal on Growth Performance and Meat Quality in Ducks. Animals.

[60] Dotas V., Bampidis V.A., Sinapis E., Hatzipanagiotou A., Papanikolaou K. (2014). Effect of dietary filed pea (*Pisum sativum* L.) supplementation on growth performance, and carcass and meat quality of broiler chickens. Livestock Sci..

[61] McNeill L., Bernard K., Macleod M.J. (2004). Food intake, growth rate, food conversion and food choice in broilers fed on diets high in rapeseed meal and pea meal with observation on sensory evaluation of the resulting poultry meat. Brit. Poult. Sci..

[62] Wiseman J. (2006). Variations in starch digestibility in non-ruminants. Anim. Feed Sci. Technol..

